# Odontography; or a Treatise on the Comparative Anatomy of the Teeth; Their Physiological Relations, Mode of Development, and Microscopic Structure, in the Vertebrate Animals

**Published:** 1845-10

**Authors:** 


### ART. VIII.

1. *Odontography; or a Treatise on the Comparative Anatomy of the Teeth; their Physiological Relations, Mode of Development, and Microscopic Structure, in the Vertebrate Animals.* By RICHARD OWEN, F.R.S. Correspondent of the Royal Academy of Sciences in Paris, Berlin, &c. &c., Hunterian Professor to the Royal College of Surgeons, London. —*London,* 1840-45. Royal 8vo, pp. 656. With 168 Plates.

2. *Report on the Microscopic Structure of Shells.* Part I. By WILLIAM B. CARPENTER, M.D. F.R.S., Fullerian Professor of Physiology in the Royal Institution of Great Britain. (*From the Report of the British Association for the Advancement of Science for* 1844.) 8vo, pp. 24. With 20 Plates.

3. *Observations on the Structure of the Shells of Molluscous and Conchiferous Animals.* By JOHN SCOTT BOWERBANK, F.R.S. &c. (*From the Transactions of the Microscopical Society,* Vol. I.)—*London,* 1844. Royal 8vo, pp. 34. With 5 Plates.

THE completion of the truly splendid work at the head of our list, of which the First Part was noticed by us at the time of its publication (vol. X, p. 208), gives us the opportunity we have long wished, of bringing under the notice of our readers, in some detail, the highly-interesting results of the laborious researches in which Prof. Owen has been so long engaged; researches whose actual merit can only be estimated by those who have penetrated somewhat deeply into the mysteries of anatomical science; but whose general results can be made appreciable by those who are obliged to content themselves with a more superficial glance. It is to this latter class that we now address ourselves. *Non cuivis contigit adire Corinthum.* Those who are seeking their daily bread amid the toils of a laborious profession, can scarcely be expected to master the details of an investigation like the present; yet we would fain hope that their youthful aspirations are not so completely quenched, as to lead them to look without interest upon its general results, especially upon such as have a direct bearing upon long-disputed questions of physiological science, or open a new field for speculation and research. To those who make the study of anatomy their main object, we say most unhesitatingly,—do not be satisfied with our sketch, but acquire the work itself,—and having acquired it, master its general principles, familiarize yourselves with the beautiful delineations which accompany it; and then, if you do not follow the author through all the minutiæ of generic descriptions, whose chief value must be to the zoologist and palæontologist, we are confident that you will experience a rich treat in accompanying him from class to class, and from order to order, and in witnessing the marvellous exactitude of the indications of affinity or diversity between the several groups, which are so frequently afforded in the single characters presented by the intimate structure of their teeth. We think, too, that the scientific public owes a debt of gratitude to the enterprising publisher, without whose aid this truly national work might never have made its appearance, at least in its present form; and we trust that, having now arrived at its completion, its sale may be such as at least to remunerate him for his large outlay.

Like too many other treatises published in Parts, Prof. Owen's Odontography has not fulfilled the promises made at its commencement; and we deem it right to allude to the circumstance, that we may not appear to exercise any partiality, by holding up certain offenders to reprobation, and letting others pass scot free. The facts of the case are these. The First Part was published in April 1840, with the following announcement: "This work will appear in Three Parts, and will be illustrated by upwards of 150 plates, to be completed in the course of the present year." The Second Part did not appear until May 1841; and the Third was then announced for 1842. Three years more passed away, however, before its appearance; the date of its publication being June 1845. It is but fair, however, to Prof. Owen that we should add, that we believe him to have more excuse for this delay than most other similar transgressors. In the first place, the work is entirely original; scarcely a single fact could be stated on the authority of another: and his personal labour was required to complete every portion of it. Further, the duties of his situation occupy a large part of his time, and these are liable to be frequently increased by unexpected occurrences. Thus since the appearance of the First Part in 1840, Prof. Owen has been called upon to prepare not merely four quarto volumes of catalogues of the Hunterian Museum, but monographs of those two extraordinary animals, the *Mylodon* and the *Dinornis*; and he has been in a manner compelled by the British Association, to undertake the laborious duty of drawing up reports on the British fossil mammalia and reptiles. Besides these works, which would seem to us quite enough for any half dozen other men, he has superintended the publication of his Hunterian Course of Lectures on the Invertebrate Animals; and has contributed not a little to the valuable Sanitary Reports of the Health of Town Commission, of which he was a *working* member. Hence we cannot but look at his default, in regard to time, with a lenient eye; more particularly as the parts of the work first published have not *grown old* in the interval, which is too frequently the case in similar circumstances. In fact, Prof.

Owen had so nearly exhausted the subject of the teeth of fishes and reptiles in his two former parts, that scarcely anything has been added by subsequent inquirers; and they are consequently as much up to the mark, as if they were published yesterday. The *present* buyers of the treatise, therefore, have no ground for complaint; and those who purchased the Parts as they appeared, had the satisfaction of possessing a work complete in itself, so far as it had extended.

Of the two pamphlets whose titles we have given above, the first embraces the commencement of an inquiry, suggested by the success of Prof. Owen's researches, into the *comparative structure* of the shells of mollusca, &c.; whilst the second contains the results of some valuable researches into the *origin and formation* of shell. We think that our readers may be interested in a brief notice of these inquiries, as a pendant to the account of Prof. Owen's labours, with which we shall now proceed.

Having given in our Eighth Volume (p. 158) a condensed summary of the history of Odontology up to the year 1839, we shall not again traverse this ground; but shall content ourselves with remarking that, notwithstanding the rapid extension of our knowledge of the *structure* of teeth, and the glimpses which had been at that time perceived, of the zoological importance of the *varieties* of structure which presented themselves in different classes, nothing positive was known in regard to the most important point in the development of teeth,—namely, the *origin* and *mode of formation* of the Dentine or Ivory. By most anatomists of recent times, as is well known, this substance was regarded as an *excretion* from the surface of the pulp; and although it had been not unfrequently spoken of by others, especially of older date, as the *ossified pulp*, no satisfactory demonstration of the *conversion* of the cells of the pulp into the solid but tubular ivory had been made public at that period. The first clear indication of a return to the more ancient doctrine, that "the dentine is the ossified pulp," is to be found in the 'Mikroskopische Untersuchungen' of Schwann, published in 1839; but he evidently felt much difficulty on the subject, in consequence of his unfortunate selection of subjects for examination. After a careful examination of the history of the controversy which took place in 1840, between Prof. Owen and Mr. Nasmyth, we have no hesitation in giving it as our decided opinion, that Prof. Owen was the first to describe unequivocally and demonstratively the history of the conversion of the pulp into ivory;—this description being contained in his memoir presented to the French Academy in December 1839, of which the general results were immediately published in the 'Comptes Rendus.' To these observations we shall presently revert; but our first duty will be to put our readers in possession of the chief elementary forms of dental structure, which Prof. Owen has encountered in the extended examination, whose details are contained in the work before us, and a general account of which is embodied in its Introduction.

The *first* and most important of these elementary structures, is that which has been usually denominated ivory, but to which Professor Owen has given the more appropriate designation of *Dentine*. Many teeth are altogether made up of it; and there are none in which it is wanting. In what may be regarded as its *typical* condition,—that in which it appears to attain its highest development, and departs most widely from other structures,—it is *unvascular;* and it then consists of a substance, which ordinarily appears homogeneous or nearly so, and which is traversed by numerous minute tubuli, running nearly parallel to each other. Where the dentine is unvascular, these tubuli run for the most part in an uniform direction; radiating from the central pulp-cavity towards the surface of the tooth. In this course, the tubes describe two, three, or more curvatures, appreciable by a low magnifying power; these are termed by Prof. Owen the "primary curvatures." With a higher power, the tubes are seen to be bent throughout the whole of their flexuous course, into minute and equal oblique undulations or gyrations, of which Retzius counted 200 within the space of 1-10th of an inch; these are termed by Prof. Owen "secondary curvatures" or gyrations. Both the primary and secondary curvatures of one tube are usually parallel with those of the contiguous tubes; and from the radiated course of these tubes, they occasion the appearance of lines running parallel with the external contour of a tooth; for when the surface of a longitudinal section is viewed with the naked eye, the light is differently reflected from the different parts of the oblique secondary curves of the tube on which it falls; but the curves being parallel to each other, and to the superficial contour of the section, they appear like the cut edges of a series of parallel and super-imposed lamellæ. The main calcigerous tubes very frequently send off ramuli, which anastomose with each other; and the intertubular substance is often seen to contain cells or cavities, with which these branches communicate. In many teeth, moreover, and especially in the tusks of the elephant, these intertubular cells (which may be regarded as dilatations of the ramuli just noticed) are disposed in lines which are parallel to the coronal contour of the tooth; hence another cause of the appearance of concentric lamellæ, and of the actual decomposition of such teeth into super-imposed lamelliform cones,—facts which have been adduced in support of the "excretion-theory" of the formation of dentine.

There is another form of dentine, however, which is intermediate in its structure between the preceding and true bone; this, which is called by Prof. Owen *vascular* dentine, is characterized by the prolongation or persistence of cylindrical canals of the pulp-cavity, in the dentinal tissue. In mammals and reptiles, these canals, which are evidently analogous to the Haversian canals of bone, are straight and more or less parallel with each other; they bifurcate, though rarely; and when they anastomose, as in the Megatherium, it is by a loop at or near the vascular dentine. Under this form, the vascular dentine is the principal constituent of the teeth of the sloth tribe. It is in the teeth of fishes that we find this structure most abundant, and presenting its most characteristic forms; the distinction between the dentinal and osseous tissues being through its intermediation gradually effaced. We there find the medullary canals of the vascular dentine, though in some instances straight and parallel and sparingly divided or united, yet in general more or less bent, frequently and successively branched, and the subdivisions blended together in so many parts of the tooth, as to form a rich reticulation. The calcigerous tubes sent off from the interspaces of the network, partake of the irregular character of the canals from which they spring, and fill the meshes with a moss-like plexus. Not unfrequently the medullary canals of the tooth are directly continuous with the Haversian canals of the bone on which it is implanted —thus demonstrating the relation between them; and there is then no central pulp-cavity.

Another modification of dentine, analogous to the preceding in its vascular character, but differing from it in the presence of radiated cells, is found in the interior of the teeth of certain reptiles, as the Iguanodon, Hylæosaurus, and Icthyosaurus, and of those of a few of the mammalia, as the Cachalot. It has been uniformly described by the authors who have observed it, as Cuvier and Conybeare, as the result of the *ossification* of the residue of the pulp; which usually remains soft, but which is here calcified, rendering the centre of the tooth solid. In this account, they were not far wrong; for the tissue in question approaches, in the combined presence of medullary canals and calcigerous cells, as closely to that of the skeleton of the species in which it occurs, as the reticulate modification of the vascular dentine in the teeth of fishes does to the osseous tissue of *their* skeleton.

The *second* primary element of tooth-structure is the *cementum*, formerly called "crusta petrosa," which corresponds in all essential particulars with bone; possessing its radiated cells in a highly characteristic form; and being also traversed by vascular canals, wherever it occurs of sufficient thickness, as in the teeth of Ruminants. This structure was first determined by Purkinjé; and he discovered it, not merely in the compound teeth of the horse and ruminant animals, but also in the simple teeth of man and the carnivora, where it forms a layer investing the fang of the tooth, and increasing in thickness as it approaches the apex of the fang. Purkinjé also observed in one instance, that this bone-like substance was continued over the enamel of the crown of a human incisor. Prof. Owen has confirmed this fact, as regards the human teeth and the simple teeth of many mammals and reptiles; the layer of coronal cement varies in thickness, being of extreme tenuity in the teeth of man and the quadrumana. The difference between the simple and compound teeth is thus proved to depend, not, as formerly supposed, on the presence of a third additional substance in the latter, but on its greater abundance and different disposition in the tooth:

"It is the presence of this osseous substance, which renders intelligible many well-known experiments of which the human teeth have been the subjects; such as their transplantation and adhesion into the combs of cocks, and the establishment of a vascular connexion between the tooth and comb; the appearances which the Hunterian specimens of these experiments present, and of the reality of which Prof. Müller satisfied himself during his visit to London, are no longer perplexing, now that we know that the surface of the tooth, in contact with and adhering to the vascular comb, is composed of a well-organized tissue, closely resembling bone.

"This correspondence of the cement, which, when it exists in sufficient quantity, becomes almost identity, with true bone, is illustrated by the varieties of microscopic structure which the cement presents in different classes of animals, and which always correspond with the modifications of the osseous tissue of the skeleton in those animals; thus the cement in the osseous fishes, in which the bone is not characterized by the radiated calcigerous cells, likewise ceases to present that character; and in reptiles and mammals, in which the radiated cells are present in the bone of the skeleton and in the dental cement, there is a close conformity as to their size and shape in both tissues.

"The most remarkable modification of mammalian cement is presented by the thick layer of that substance which invests the molars of the extinct megatherium; besides abounding in calcigerous cells, it is here traversed by straight, parallel, and occasionally bifurcated medullary canals, arranged with regular intervals, and directed from the exterior of the tooth somewhat obliquely to the surface of the unvascular dentine, close to which they anastomose by loops, corresponding with, and opposite to, those formed by the medullary canals of the vascular dentine of the same tooth." (Introduction, p. xxi.)

Under every modification, the cementum is the most highly organized and most vascular of the dental tissues; and its chief use is to form the bond of vital union, between the denser and commonly unvascular constituents of the tooth, and the bone in which the tooth is implanted. In the herbivorous mammalia, it not only invests the exterior of the teeth, but penetrates their substance in vertical folds, which vary in number, form, extent, thickness, and degree of complexity; and these contribute to maintain that inequality of the grinding surface of the tooth, which is so essential to its function as an instrument for the comminution of vegetable substances. A similar structure has been found by Prof. Owen in the teeth of a few reptiles now extinct.

We now come to the *third* and last of the primary elements of tooth-structure; namely, the enamel. This is the least constant of the dental tissues; it is more frequently absent than present in the teeth of the class of fishes; it is wanting in the entire order Ophidia among reptiles; and it forms no part of the teeth of the Edentata and many Cetacea among mammals. Moreover its structure is by no means constant, even where its presence is evident. Like dentine, it presents several shades of variety. Its highest or most specialized condition is presented in the class mammalia; whilst in fishes it frequently approaches so closely to dentine in its microscopical and chemical characters. We shall first describe its most distinctive form, such as it presents in mammals. The enamel of the molar tooth of a calf, which has just begun to appear above the gum, and which can readily be detached from the dentine (especially near the commencement of the fangs), is resolvable into apparently fine prismatic fibres; if these fibres be separately treated with dilute muriatic acid, and the residue examined, with a moderate magnifying power, in distilled water, or, better in dilute alcohol, portions of more or less perfect membranous sheaths or tubes will be discerned, which inclosed the earthy matter of the minute prism, and served as the mould in which it was deposited. When, however, the enamel is examined in a tooth that has been completely formed and in use, the small proportion of animal matter which then remains does not yield any indication of its primitive organic structure; and the only information it will then afford is derived from the microscopic examination of sections and fractured surfaces. It is then ascertainable, that the layer of enamel consists of an assemblage of prismatic fibres closely impacted together, their form being more or less regularly hexagonal; they are directed nearly at right angles to the surface of the dentine; and their central or inner extremities rest in slight but regular depressions on the periphery of the coronal dentine, whilst their free extremities form the surface of the enamel, By examining the latter with reflected light, under a linear power of 300, they are seen to present very much the appearance of the face of a honeycomb. In the human tooth, the fibres which constitute the masticating surface are perpendicular or nearly so to that surface; while those at the lower part of the crown are transverse to the axis of the tooth, and consequently have a position best adapted for resisting the pressure of the contiguous teeth. The strength of the enamel-fibres is further increased by the graceful wavy curves in which they are disposed; these curves are in some places parallel, in others opposed; their concavities are commonly turned to each other, where the shorter fibres, which do not reach the exterior of the enamel, rest by their gradually-attenuated peripheral extremities upon the longer fibres. Other shorter enamel-fibres extend from the outer surface of the enamel towards the dentine, and are wedged into the interspaces of the longer fibres. When the enamel-fibres are examined with a high magnifying power, they are seen to be marked with a series of minute and closely-set transverse striæ, along the whole or a part of their length; this appearance is wanting, however, in the enamel of *fossil* mammalian teeth; or at least in those specimens examined by Prof. Owen. He does not here attempt to give any explanation of them, but contents himself with confirming the remark of Retzius, that they probably belong to the capsule and not to the enamel-fibre; we shall have occasion to notice them again, when speaking of the development of the enamel. All his researches confirm the statement of Schwann, in regard at least to the enamel of mammalian teeth, that it consists of a layer of prismatic *cells* in close apposition with each other; the extremities of which constitute the free and attached surfaces of the enamel, whilst their length corresponds for the most part with the thickness of the layer. We shall hereafter see that Dr. Carpenter and Mr. Bowerbank have discovered a structure in certain shells, which exactly coincides with this form of enamel, even to the presence of the transverse striæ; but which is on so much larger a scale, that the prisms, which in the enamel of the horse are about 1-5600th of an inch in diameter, are sometimes as much as 1-100th of an inch across.

This condition of the enamel, like the corresponding one of the mammalian dentine, whilst it distinguishes that tissue from the true osseous texture, and perfects it for its mechanical application, at the same time removes it from the influence of the conservative and reparative powers of the living organism. When once formed and exposed, the mammalian enamel is less able than the other structures to offer any resistance, by its vital endowments, to the influence of the external decomposing forces; but this inferiority is amply compensated by its superior mechanical endowments. Containing not more than 2 or 3 per cent. of organic matter, and having the calcareous elements so disposed as to possess the greatest cohesion, the enamel is by far the firmest and most durable of the dental structures: and accordingly we find it disposed in situations where it may offer the most advantageous resistance. In most simple teeth, it is limited to the "crown" of the tooth, where it caps over the dentine. It sometimes, however, forms only a partial investment to the crown; as in the molar teeth of the Iguanodon, the canine teeth of the hog and hippopotamus, and the incisors of the Rodentia. In these, the enamel is placed only on the front of the tooth, but is continued along a great part of the inserted base, which is never contracted into one, or divided into more fangs; so that the character of the crown of the tooth is maintained throughout its extent, as regards both its shape and structure. The partial application of the enamel in these "dentes scalprarii" operates in maintaining a sharp edge upon the exposed and worn end of the tooth, precisely as the hard steel keeps up the outward cutting edge of the chisel, by being welded against an inner plate of softer iron. In the herbivorous mammalia, with the exception of the Edentata, vertical folds or processes of the enamel are continued into the substance of the tooth; varying in number, form, extent, and direction; and producing, by their superior density and resistance, the ridged inequalities of the grinding surface, on which its efficacy in the trituration of vegetable substances depends.

In fishes, however, the enamel frequently bears so close a resemblance to the dentine, as scarcely to be distinguishable from it. The fine calcigerous tubes are present in both substances, and undergo similar subdivisions; the directions only of the trunks and branches being reversed, in conformity with the contrary course of their respective developments,—the matrix of the enamel being internal, whilst that of the dentine is external. The proportion of animal matter is also greater in the teeth of fishes, than it is in that of higher vertebrata; and the proportion of the calcareous salts incorporated with the animal constituents in the walls of the tubes, is greater as compared with the sub-crystalline part deposited in the tubular cavities. In reptiles, the proportion of the hardening salts, and consequently the density of the enamel, are increased; but the course, size, and ramification of the calcigerous tubes still bear considerable analogy to those of the dentine; whilst the prismatic form of the enamel-cells, and their minute striations, which constitute the characteristic features of the enamel in the mammalian class, are not present in that tissue in the cold-blooded vertebrata.

It has long been recognized as a general law, that the higher an animal is placed in the scale of organization, the more distinct and characteristic are not only the various organs of the body, but the different tissues which enter into their composition. We have now seen how well that law is exemplified in the teeth; although in the comparison of these organs we are necessarily limited to the range of a single primary group of animals. We have seen, for example, that the dentine is scarcely distinguishable from the tissue of the skeleton in the majority of fishes; but that its peculiarly dense, unvascular, and resisting structure, which is the exceptional condition in fishes, is its prevalent character in the teeth of the higher vertebrata. And the enamel, whose characters in mammalia are so peculiar, sinks, in fishes, into a condition in which it can hardly be called a special form of tooth-structure.

We now pass on to consider the development of the teeth, to which John Hunter was the first to direct particular attention. It was by him that three different parts of the matrix were first distinguished, as destined for the production of three different kinds of primary tissue now described; the several parts of this matrix—termed by Prof. Owen respectively the "dental pulp," the "enamel pulp," and the "capsule" or "cemental pulp"—being first distinctly indicated in the 'Natural History of the Human Teeth.' But no definite account was given by Hunter, either of the part which each formative organ plays in the development of its corresponding tissue, or of the development of the matrix itself. The latter subject has been chiefly elucidated by the observations of Arnold, Purkinjé and Raschkow, Valentin, and Goodsir, of which we gave some account in our former article (vol. VIII, p. 165 et seq.;) but as we were at the same time obliged to point out the important discrepancies existing between their statements, we shall here introduce a notice of the results of Prof. Owen's more extended researches, which, as will be seen, bear a close correspondence with those of Mr. Goodsir.

"The dentinal pulp is always the first developed part of the matrix, and makes its appearance in the form of a papilla, budding out from the free surface of a fold or groove of the mucous membrane of the mouth, and generally of that which covers the inner side of the jaws or their rudiments. In certain fishes, as the shark, the tooth is completed without the development of the matrix proceeding beyond this 'papillary' stage.

"The first papilla may be distinctly recognized in the maxillary mucous groove of a human embryo, one inch in length; the others quickly follow. By the growth of the contiguous mucous membrane, the papilla appears to sink into a follicle; and by the development of three or four lamellar processes from opposite sides of the mouth of the follicle, and their mutual cohesion, the papilla is inclosed in a capsule; this 'capsular' stage of development is completed in the human fœtus at the fifteenth week. The capsule is the part of the matrix destined for the development of the cement. In many fishes and in serpents, the teeth are completed without the development of the matrix proceeding beyond this stage.

"In those teeth which are defended by enamel, a pulp destined for its production is developed from the inner surface of the capsule, opposite to that which the dentinal pulp is attached. In the human subject, the 'enamel pulp' makes its appearance as a soft gelatinous substance adhering to the opercular plates closing the capsule, and the adjoining inner surface of the capsule, at the sixteenth week; the surface of adherence of the 'enamel pulp' is progressively extended until it is separated by a mere linear interspace from the base of the 'dentinal pulp.' Whatever eminence or cavities the one has (says John Hunter,) the other has the same, but reversed; so that they are moulded exactly to each other." (Introduction, p. xxix.)

In regard to the formation of the dentine from the dentinal pulp, it is well known that two distinct views have been entertained. One of these, which may be termed the "excretion-theory," is generally regarded by physiologists as having had Hunter for its parent; although he does not express himself very clearly on the subject. This theory supposes that the pulp stands to the tooth-bone in the relation of a gland to its secretion; that the formative virtue of the pulp resides in its surface only; that the dentine is deposited upon and by the formative or secretive surface of the pulp, in successive layers; and that the pulp, exhausted, as it were, by its secretive activity, diminishes in size as the formation of the tooth proceeds, —except in certain species, in which the pulp is persistent, and maintains an equable secretion of dentine throughout the life-time of the animal. This opinion regarding the formation of dentine was most clearly set forth by Cuvier; who regarded himself as confirming, in this particular, the views of Hunter.

The other theory,—which now indeed can scarcely be any longer termed such, since it is an established fact,—has been known as the "conversion-theory." On this view, the cells of the pulp are actually converted, by a series of metamorphoses hereafter to be explained, into the tubular dentine; which is therefore to be regarded, not as a new formation from the surface of the pulp, but as neither more nor less than the calcified pulp itself; hence those cases, in which the formation of new dentine is continually going on at the base of the tooth, are to be regarded as simply examples of the continued growth and metamorphosis of the pulp, which processes are elsewhere limited. It is to Purkinjé and Raschkow that we owe the earliest advance towards this discovery; these observers having been the first, it would appear, to submit to careful microscopic examination the structure of the pulp prior to the formation of the dentine, and to endeavour to trace the changes which it undergoes during that process. As we have noticed their researches, and those subsequently made by Schwann, on two former occasions (vol. VIII, p. 166, and vol. IX, p. 514,) we shall here only give Prof. Owen's summary of their results, which we believe to be perfectly correct.

"The main facts, then, which may be considered as established by the researches of Purkinjé and Schwann, relative to the formation of dentine and the changes which the dentinal pulp undergoes during the process, are the following. The proper tissue of the pulp consists of minute nucleated cells with capillary vessels and nerves, invested by a dense structureless membrane (membrana preformativa), which disappears during the formation of the dentine. The superficial pulp-cells assume an elongated form; they correspond in diameter and direction with the tubes of the contiguous cap of dentine. These or similar cells are observed, in a state of transition into dentine, in the interspaces between the pulp and the previously-formed cap of dentine; they adhere to the latter when it is displaced from the pulp." (Introduction, p. xxxviii.)

The chief points that remained to be determined were the relation of the dentinal pulp to the transitional cells between it and the dentine; the nature of the transition; and the relation of the cells to the dentinal tubes and the intertubular tissue. By Purkinjé and Raschkow it appears still to have been thought, that the pulp supplied the material only for the production of dentine; and that this was deposited in successive strata between its surface and the preformative membrane. On the other hand, Schwann evidently leaned to the ancient doctrine that the dentine is the ossified pulp; but the nature of the subjects selected by him for his observations, and also by Mr. Nasmyth who early followed in the same track, was not favorable for the detection of the real character of the process, for reasons thus stated by Prof. Owen:

"The shape of the teeth of the mammalia selected by them for examination, will not yield a view of the cap of new-formed ivory and the subjacent pulp, in undisturbed connexion, by transmitted light, with the requisite magnifying power; and if placed under the microscope as an opake object, the light is reflected from the cap of ivory, and displays only the characters of its surface, and not its relations to the surface of the pulp in contact with it. To examine this surface microscopically, either in a human tooth, or in that of any of our domestic quadrupeds, the cap of dentine must be removed; and the exposed surface of the pulp, and the corresponding surface of the dentine, must be examined as opake objects by reflected light. Or, if the layer of the dentine be then enough to allow the transmission of sufficient light, it must be removed from the subjacent pulp before it can be so examined. It is, therefore, obvious that any inference as to the structure of the pulp's surface, or the nature of its previous connexion with the transitional cells and the superincumbent layers of dentine, which may be founded on appearances observed under the circumstances above mentioned, is liable to the objection that the natural relations of the parts observed have been destroyed. If the dentine be the ossified pulp, as Dr. Schwann was disposed to believe, then the calcified part of the growing tooth has been violently displaced from the uncalcified part; and the part of the pulp which thus presents itself is a lacerated and not a natural surface.

"But to the observer who regarded the dentine as a secretion from the pulp's surface, every modification which he might detect on the surface after the displacement of the dentine, would appear natural, and perhaps be described as such with the view to the elucidation of the secreting process. Thus the cells which might be observed in progress of ossific transition into dentine, would appear as independent parts, and the products of a secreting property; their detached condition being, all the while, a necessary result of the artificial displacement of the new formed cap of ivory, and the consequent laceration of the pulp's surface. In the terms of the 'excretion-theory' the exposed surface of the pulp over which the cells lie scattered is a 'formative surface;' the nucleated cells are naturally 'detached,' and the ivory or dentine resulting from their calcification and metamorphosis is, in respect to the pulp, 'altogether a distinct formation, and by no means an ossification of the pulp." (Introduction, p. xl.)

That such were, in fact, the deductions of an acute observer, from researches analogous to those of Purkinjé and Schwann, makes it evident that the doctrine of "conversion," to which the latter manifested a leaning, could not be regarded as by any means established, whatever amount of *a priori* probability it might be considered as possessing. The first *demonstration* of the organic continuity of the cap of dentine with the supporting vascular pulp, was afforded to Prof. Owen by the examination of the thin, lamelliform, transparent teeth of a fœtal shark; in which the various stages of the conversion of the pulp into dentine could be observed, without any disturbance of their natural relations,—a condition evidently of the greatest importance. By his observations on this fortunate *subject*, Prof. Owen was able to attain that clear idea of the nature of the development of dentine, which is expressed in his 'Theory of dentification by centripetal calcification of the pulp's substance,' submitted to the French Academy, in December 1839. Of the truth of this theory, he has since obtained evidence from widely different sources; and we cannot doubt that it is in the main correct, although some of the details may possibly require slight modification, as our knowledge of the general history of cell-growth and metamorphosis becomes more precise. We shall, therefore, endeavour to put our readers in possession of it, by an abridgment of Prof. Owen's descriptions;—premising, however, that we cannot make them as intelligible as we could wish, without the aid of figures.

The essential character of each of the three formative organs already noticed, is *cellular;* but the cells differ in each organ, and derive their specific characters from the properties and metamorphoses of their *nucleus;* upon which the specific microscopical characters of the resulting calcified substances depend. In the cells of the "dentinal pulp," the nucleus fills the parent-cell with a progeny of nucleoli, before the work of calcification begins. In the "enamel-pulp," the nucleus of the cell disappears, like the cytoblast of the embryo-plant in the formation of most vegetable tissues. And in the cells of the "capsule" or "cemental pulp," the nucleus neither perishes nor propagates, but retains its individuality, and gives origin to the most characteristic feature of the cement, viz.—the radiated cell.

The dentinal pulp, at the commencement of the calcifying process, consists of a mass of nucleated cells, imbedded in and supported by a homogeneous, minutely sub-granular, mucilaginous substance, the "blastema;" and through this is distributed a network of capillary blood-vessels, originating from the base of the papilla, together with a plexus of nerves. The capillaries present a sub-parallel or slightly-diverging pencillate arrangement during their progress through the substance of the pulp; but they preserve their looped and reticulate termination near its apex. The accompanying nerves terminate in loops. The cells, which are smallest at the base of the pulp, and have large simple sub-granular nuclei, soon fall into linear series directed towards the periphery of the pulp; and where the cells are in close proximity with that periphery, they become more closely aggregated, increase in size, and present a series of important changes in their interior. A pellucid point appears in the centre of the nucleus, which increases in size, and becomes more opake around it. A division of the nucleus in the course of its long axis is next observed. In the larger and more elongated cells, still nearer the periphery of the pulp, a further subdivision of the nuclei is observed, in a transverse as well as in a longitudinal direction; and the subdivisions become elongated, with their long axes vertical, or nearly so, to the plane of the pulp and to the field of calcification. At this stage, Prof. Owen's figure represents each cell as possessing several distinct nuclei, arranged in rows; these being formed by the longitudinal and transverse division of the original single nucleus; and the rows pointing from the centre towards the periphery of the pulp. The subdivided and elongated nuclei become attached by their extremities to the corresponding nuclei of the cells in advance; and the attached extremities become confluent; so that lines or files of nuclear matter are formed, which present an unbroken continuity from one cell to another.

While these changes are proceeding, the calcareous salts of the surrounding plasma begin to be accumulated in the interior of the cells, and to be aggregated in a semi-transparent state about the central granular part of the elongated nuclei, which now present the character of rows of minute secondary cells; and the salts occupy, in a still clearer and more compact state, the interspaces of such cells. The rows of secondary cells remain uncalcified in the midst of the solid calcareous deposit, and thus constitute the *tubuli* of the dentine; in which a granular or moniliform aspect may generally be traced; and in which, too, it would appear that some scattered deposit of calcareous matter takes place. Around the tubes, in a transverse section, is a small circular space, manifestly distinct from the intertubular substance; and this is regarded by Prof. Owen as the indication of the membrane surrounding the elongated and coalesced nucleoli or secondary cells. The indications of the primitive boundary or proper parietes of the parent cell are commonly lost; but they are sometimes very distinctly preserved, especially in certain species of the mammalian class. They are figured by Prof. Owen in the molar of the Mylodon, in the incisive tusk of the Dugong, in the premolar and canine of the Pteropus, in the incisor of the Chimpanzee and of Man, and in the molar of a Rhinoceros. Of the correctness of some of these delineations, we have been able to satisfy ourselves by personal observation; and we cannot hesitate, therefore, in ascribing to Prof. Owen the merit of first detecting, in the completely-formed dentine, the original cells of the pulp. When a layer of the calcified cells is carefully detached, the exposed uncalcified surface of the pulp presents the appearance of a network; the meshes being formed by the exposed cells, and the intervening very thin layer of blastema. Each mesh shows, by well-directed light under a high power, several points, each of which is the centre of one of the meshes of a finer network; and these points are the ends of the granular elongated nuclei, which have been torn from the cavities of the dentinal tubes in the displaced cap of dentine. The adhesion of the primary cells appears to be greater longitudinally than laterally, so as to give a tendency to that fibrous appearance, which the fractured surface of dentine usually presents; and this adhesion is strengthened by the continuity of the secondary cells or elongated nuclei. These usually uniting with each other at obtuse angles, and not in a perfectly straight line, form the "secondary gyrations or curvatures" of the tubuli. The primary curves depend upon the arrangement of the primary linear series of parent-cells.

Hence we are to regard the dentine as composed of the cells of the pulp, which have become consolidated by the deposition of calcareous matter, and which have coalesced, more or less completely,—generally even to the entire obliteration of the original partitions; the tubuli of the dentine are formed by a series of metamorphoses from the nuclei of those cells; whilst the intertubular substance is the result of the secretion that fills up the cavity of the parent cell, saving in those portions which are occupied by the tubuli. In some animals, as already stated, it is not uncommon to find minute cells, in this intertubular substance, communicating with the ramuli of the tubes, of which indeed they may be considered as dilatations; these cells, to which attention has been particularly directed by Mr. Nasmyth, are probably to be regarded in the light of secondary cells, having their origin as offsets from the linearly-arranged nuclei of the primary, but more fully developed than are those which by their coalescence form the tubuli.

As the pulp becomes calcified from its exterior towards its interior, it progressively decreases; fewer nuclei are developed in the cells, and these do not acquire so large a size. The diminution in both respects proceeds unequally, however, in the cells of the same stratum. Here and there, the linear tract formed by the nuclear matter, in a part of a smaller calcifying cell containing fewer nuclei, may be observed to unite with the converging extremities of *two* residuary tracts, or areæ of dentinal tubes, of a calcified cell in advance. It is thus that the *bifurcation* of the tubes is produced; and a repetition of this confluence, which becomes more frequent as the calcifying process approximates the centre and base of the pulp, gives rise to the dichotomous divisions of the main tubes. In some of the cells at and near the central and basal part of the pulp, the nucleus has undergone no division; but has become merely elongated, and sometimes angular or radiated. In others it has altogether disappeared. This altered mode of action or change in the nuclei of the smaller central cells of the pulp, is the first and essential step in that modification of the dentinal tissue, which produces the substances termed by Prof. Owen "osteo-dentine" and "vaso-dentine." In the former, many of the cells retain their nucleus undivided, and the hardening salts are impacted around it in the interior of the cell; and the more or less radiated or stellate form, which the nucleus acquires by the prolongations it sends forth, produces a certain resemblance to bone. In the production of "vaso-dentine," many of the cells lose their nucleus, which seems to have become dissolved. In both the latter modifications of dental tissue, the blood-vessels remain, and establish the wide tubular tracts in the calcified substance, to which the name of vascular canals is given. In true, hard, or unvascular dentine, no trace of the blood-vessels remains; and any changes which it undergoes, subsequently to the process of calcification, must be the result of the endowments of its component cells, and of the rows of nucleoli or partially-developed secondary cells, which fill the dentinal tubes. It will naturally be asked how, upon the "conversion-theory," the withdrawal (so to speak) of the capillary blood-vessels from the vascular pulp takes place, during the process of calcification? About this, however, no real difficulty exists; for it is just as easy for the capillary loops to retreat, as it was for them to advance in the first instance; and a similar process takes place in several other cases. Prof. Owen describes the extremities of the capillaries, at the part where calcification has commenced, as being in a state of congestion, and crowded with blood-discs, which are pressed together into polyhedrons, and are apparently stagnated and left out of the current of the circulation. These blood-discs are evidently in a state of metamorphosis, the degree of which varies according to their proximity to the calcified dentine; those nearest to it being distinguished by their mulberry form, due to the development of contained granules, by an increase of size, by a change from the circular to the elliptical form, and by a gradual loss of the characteristic colour, which is longest retained by the central granular matter. The tunics of the capillary vessel, containing these altered blood-discs, become gradually attenuated, and disappear as if dissolved, before reaching the field of conversion; and (although we are not so informed by Prof. Owen) we presume that the same change happens to these corpuscles, whose office it probably is to select and assimilate the materials which are to be employed in the process of calcification,—like the labourer who mixes and tempers the mortar, which is to be employed by the mason in his constructive operations. "The open mouths of the central last-formed ends of the calcified dentinal tubes," says Prof. Owen, "are always ready to receive the plasma transuded from the capillaries remaining in the uncalcified portion of the pulp, or in those tracts of it which constitute the vascular canals;" or, we would add, to take up the nutrient material supplied by the liquefaction of the assimilative cells just described.

It will be obvious from the preceding detail, how great is the importance of the *nucleus* of the cell, in its subsequent transformations; and here Prof. Owen's observations fully coincide with those, to which we have recently drawn the attention of our readers in other quarters.

We next proceed to consider the production of the enamel; the matrix of which differs from the dentinal pulp at its first formation, by the more fluid state of its blastema, and by the fewer and more minute cells which it contains. The source of this fluid blastema appears to be the free inner vascular surface of the capsule; in fact, we have been in the habit of regarding the cells of the enamel-pulp as constituting the epithelium that we should expect to find lining the mucous follicle. When thus viewed, the enamel bears a remarkable correspondence, in character as well as in structure, with the shell-substance hereafter to be described. As the enamel-blastema approaches the dentinal pulp, it acquires more consistence by an increased number of its granules; and it contains more numerous and larger cells, many of which show a nuclear spot, some even a nucleolus. These cells, which were at first spherical, become more closely impacted in the portion of the enamel-pulp most distant from the capsule; and their sides becoming flattened against one another, they are pressed into hexagonal or polygonal forms, the fluid blastema being now almost excluded from between them. In this condition, they very closely represent the cellular tissue of plants.—The final metamorphosis of these cells into moulds for the reception of the solidifying salts, is confined to the portion in close contiguity with the surface of the dentinal pulp. Here the cells increase in length, lose all trace of their nucleus, and become converted into long and slender cylinders, usually pointed at both ends, and pressed by mutual contact into a prismatic form. These elongated prisms have the property of imbibing the calcareous salts of the enamel from the fluid plasma, and of compacting them in a clear and almost crystalline state in their interior: the disappearance of the nucleus being evidently the condition of the absence of any permanent cavity, cell, canal, or other modification of the mineral matter,—at least in the enamel of the calf, which does not present the transverse striæ that are seen in the enamel-fibres of the human subject. These striæ may be due, in Prof. Owen's opinion, to a subdivision of the cavity of the cylinder into compartments, by a multiplication of delicate nucleoli; or by some modification of the walls of the cylinder by the remains of such multiplied nucleoli. As the calcification of the prismatic cells, by their own secreting action, proceeds, they become more and more closely impacted; and at last their membranous walls cease to be distinguished, except at the surface of the enamel next the capsule, where it is still in progress of growth. It is remarkable that the whole of the cellular basis of the enamel-pulp is not converted into the prismatic moulds of the calcareous fibres; at least in the valleys of the complex crown of the molar in the herbivorous mammals, this part of the enamel-pulp originally occupies more space than is subsequently filled by the enamel; the superfluous peripheral part seems to be absorbed, and its place to be occupied by a growth or thickening of the vascular capsule. No capillaries pass from the capsule into the cellular pulp of the enamel; and its cells must, therefore, grow and select their contents, by their own inherent powers, from the subjacent vascular membrane.

Lastly, the blastema of the capsule or "cemental pulp," consists, like the matrix of the dentine and enamel, of nucleated cells, distributed in the midst of a granular blastema, copiously supplied with blood-vessels. The process of calcification begins in the portion nearest the dentine; and consists in the absorption of calcareous matter into the cavities of the cells, and the more close aggregation of the cells to each other. If the capsule be examined during the process of ossification, it is seen that the exterior surface of the newly-formed cement is hollowed into numerous little cup-shaped cavities, in which lie the cells that are next to undergo calcification. It is from the peculiar changes which occur in the nucleus, that the character of the cementum is chiefly derived. The nucleus is large and granular, and almost fills the clear area of the cell; and as the process of calcification takes place, it sends out radiating prolongations, which extend quite to its borders. The calcareous salts which penetrate the cell, do not seem to be deposited in the space occupied by the nucleus; and thus the stellate cavities or lacunæ are left, when the nuclear matter is removed, which are so exactly analogous to those of bone, as to serve to identify the two tissues. These radiating cavities, however, although the most common characteristic of the cementum are not constant; and are notably absent in the thin layer of that substance, which originally surrounds the crown of the human teeth and of the simple teeth of the quadrumana and carnivora. The layer of the capsule, from which this is formed, consists simply of granular blastema, without nucleated cells; and the substance produced by its calcification cannot be regarded, therefore, as properly-developed cementum.

In the deep sockets of the teeth of persistent growth, the matrix is maintained by the constant additions of new blastema and cell-material, to the bases of the dentinal, enamel, and cemental pulps; and the process of conversion is continually going on.

The history of the general structure and development of the Teeth, through which we have now conducted our readers, is contained in the Introduction to the Odontography; and it remains for us to give some idea of the nature of the work itself. This consists, in fact, of a detailed account of all the principal modifications of the teeth, whether in form, arrangement, or elementary structure, which are presented in the classes of Fishes, Reptiles, and Mammals; such details cannot be abridged; and when isolated they lose much of their interest. Moreover, it is scarcely possible to make them intelligible without the accompanying plates. We shall therefore confine ourselves, for the most part, to those general observations upon the structure of the teeth in each class, which we think will be most interesting to our readers; adding, here and there, a few isolated facts which will give an idea of the curious results of the investigation.

Prof. Owen is far from asserting that the structure of the Teeth, as made manifest by the Microscope, "forms a new, distinct, and specific guide for classifying the different members of the animal kingdom, and determining their respective types." But he states "that the Teeth, by their microscopic structure, as well as their more obvious characters, form important, if not essential aids to the classification of existing, and the determination of extinct species of vertebrated animals; but in this comparatively restricted sphere, the teeth have different degrees of value, as zoological characters, in different classes; the lowest degree being in the class of Fishes, and the highest in that of Mammals." (Introduction, p. lxxii.)

The teeth of Fishes, in whatever relation they are considered,—whether in regard to number, form, substance, structure, situation, or mode of attachment,—offer more various and striking modifications than do those of any other class of animals. These organs, being here first introduced into the ascending series of animals, manifest the principle of *repetition*, which is seen in many other organs, where they make their first appearance;—as in the mouths of the polypes, or the generative organs of the tape-worm. Hence there are extremely few genera of fishes, that can be characterized by a definite dental formula, like mammals in general; and in many fishes, as the pike and silurus, the teeth crowd the mouth in such numbers as scarcely to admit of being counted. The *forms* of the teeth in this class are too diverse to admit of being included in any general description; we may mention, however, that among those which are simply conical, there are some so minute, numerous, and closely aggregated, as to resemble the pile of velvet; whilst others equally slender are elongated, so as to form bristles, which are sometimes pointed, recurved, or even barbed like a fish-hook. The pike and other predatory fishes have larger and slightly recurved conical teeth; which strongly resemble the canine teeth of carnivorous mammals. Other teeth, again, present a wedge-shaped laminated form, sometimes with sharp cutting edges, so as to have the character of a lancet; this is particularly seen in the sharks. Prof. Owen mentions a pair of jaws of the great white shark (Carcharodon), preserved in the United Service Museum, of which the upper one measures 4 feet, and the lower one 3 feet 8 inches, following the curvature; the length of the largest tooth in this specimen is 2 inches, and the breadth of its base $1\frac{3}{4}$ inch; the total length of this shark was 37 feet. Now fossil teeth, precisely corresponding in form with those of the Carcharodon, occur abundantly in the tertiary formations of both the old and new continents; some of which teeth exhibit the extraordinary dimensions of 6 inches in length and 5 inches across the base. If, therefore, the proportions of these extinct Carcharodons corresponded with those of the existing species, they must have equalled the great mammiferous whales in size; and, combining with the organization of the shark its bold and insatiable character, they must have constituted the most terrific and irresistible of the predacious monsters of the ancient deep. From the dimensions of the jaws of the recent specimen just adverted to, it is evident that its gape must have been wide enough to make no difficulty in receiving the body of a stout man; but even this is one of a degenerate race; for the sharks of former times would have "made no bones" of a grampus some 30 or 40 feet long, and 10 or 12 feet round the belly, or of a ship's long boat with its crew of the same dimensions; but would have bitten them in half as easily as we should bite a radish. Truly we may be thankful that we do not live in such times. There is no knowing what fossils may hereafter come to light; perhaps our geologists may alight some day upon a tooth, that Prof. Owen will find to have belonged to a shark large enough to have received the Great Britain into its capacious maw, and strong enough to have cut it in half, clean through the engine-room! Of a form precisely opposite to these, are the flattened crushing teeth of certain fishes; which enable them to masticate the stems of corals, or to break down the bony armour in which their prey may be encased. The surfaces of these are variously sculptured; often in a very beautiful manner.

The *situation* of the teeth is much more various in the class of fishes than it is in any of the higher animals. We cannot give any details on this subject, without giving an account of the numerous bones which enter into the composition of the mouth; but we may state generally, that whilst some fishes, as the sharks and rays, have the teeth restricted to the bones or cartilages bounding the anterior aperture of the mouth, and others, as the roach, dace, &c. have them implanted in the pharyngeal bones only, so as to surround the posterior aperture of the mouth,—others again, as the wrasse and the parrot-fish, have teeth in both these situations; whilst in many tribes we find teeth implanted on every bone which enters into the composition of the mouth, as the palatines, the vomer, the lingual bones, and the branchial arches, and (more rarely) on the transverse or pteregoid, the sphenoid, and the superior maxillary, as well as on those which surround the anterior and posterior orifices of the mouth. In the lampreys, and in one of the osseous fishes, most of the teeth are attached to the lips.

The teeth of Fishes present greater diversity in the mode, as well as in the place, of their *attachment*, than is observable in those of any other class of animals. With the exception of the anomalous teeth in the rostrum or beak of the saw-fish, and the composite dental masses of the Chimæroids, there are no teeth in fishes which can be said to be *permanent;* and among those few which are implanted in distinct sockets, the roots are nowhere divided,—that mode of connexion being peculiar to mammalia. By far the most common mode of attachment of the fully-formed teeth in the present class, is by a *continuous ossification* between the dental pulp and the jaw; the transition being gradual between the structure of the tooth and that of the bone; and the tooth, prior to the anchylosis, being connected by ligamentous substance, either to a plane surface, an eminence, or a shallow depression in the jaw-bone. This condition sometimes remains permanent, i. e. until the tooth is shed, especially in the cartilaginous fishes, as the sharks and rays. In the Lophius (angler or frog-fish), the large posterior teeth always remain moveably connected to the jaw, by highly-elastic, glistening ligaments, which pass from the inner side of the base of the tooth to the jaw-bone. These ligaments do not permit the tooth to be bent outwards beyond the vertical position, the hollow base of the tooth then resting upon a circular ridge growing from the alveolar margin of the jaw; but the ligaments yield to pressure upon the tooth in the contrary direction, and its point may thus be directed towards the back of the mouth; the instant, however, that the pressure is remitted, the tooth flies back, as by the action of a spring, into its usual erect position; the deglutition of the prey of this voracious fish is thus facilitated, and its escape prevented. In the attachment of the incisors of the file-fish, we find a curious sort of double gomphosis, the jaw and the tooth reciprocally receiving and being received by each other; the base of the tooth is hollow, and is supported (like the claws of the Felenis, or the hollow horns of the Ruminants) upon a bony prominence which arises from the base of the socket, whilst the socket in its turn embraces the base of the tooth. Generally speaking, even where the teeth of fishes are implanted in sockets, they exhibit a continuity of structure with the adjacent bone at *some* point; so that they are not the free independent organs, which exist in the mammalia. The following are two interesting cases of peculiarity in the attachment of the teeth of fishes.

"If the engineer would study the model of a dome of unusual strength, and so supported as to relieve from its pressure the floor of a vaulted chamber beneath, let him make a vertical section of one of the crushing pharyngeal teeth of the Wrasse. The base of this tooth is slightly contracted, and is implanted in a shallow, circular cavity, the rounded margin of which is adapted to a circular groove in the contracted part of the base; the margin of the tooth, which immediately transmits the pressure to the bone, is strengthened by an inwardly-projecting convex ridge. The masonry of this internal buttress and of the dome itself, is composed of hollow columns, every one of which is placed so as best to resist or transmit in the due direction the superincumbent pressure." (p. 8.)

The wrasses feed on testaceous molluscs, crustaceans, echini, &c., which they seize with their long anterior conical teeth, and crack and bruise by means of these powerful pharyngeals. Allied to these are the scari or parrot-fishes, which are adapted, by a somewhat similar conformation, to browse upon the animal matter of the stony corals that clothe, with a richly-tinted verdure, the bottom of the sea, as the ruminant quadrupeds crop the herbage of the dry land. The irritable bodies of the gelatinous polypes, which constitute the food of these fishes, retract, however, when touched, into the star-shaped cavities of their stony support; and the scari are therefore provided, on the front of their jaws (which are prominent, short, and stout,) with a dental apparatus fitted to break away this calcareous protection; whilst the posterior pharyngeal teeth, which resemble those of the wrasse, are adapted for its comminution. Again,

"In another case, in which long and powerful piercing and lacerating teeth were evidently destined, from the strength of the jaws, to master the death-struggles of a resisting prey, we find the broad base of the tooth divided into a number of long and cylindrical processes, which are implanted like piles in the coarse osseous substance of the jaw; they diverge as they descend; and their extremities bend and subdivide like the roots of a tree, and are ultimately lost in the bony tissue. This mode of implantation of the teeth, which I have detected in a large extinct sauroid fish (Rhizodus,) is perhaps the most complicated which has been yet observed in the animal kingdom." (p. 8.)

It will be observed that this case offers no exception to the general rule just stated; for although the roots are so divided, they are not implanted in a distinct socket, but become continuous with the bone.

The *substance*, also, of the teeth of fishes, presents an unusual variety. In some instances they are horny, flexible, and elastic; but in general they are composed of an osseous substance, somewhat denser than that of the jaws to which they are attached. In the flying-fish and sucking-fish, the substance of the tooth is uniform, and not covered by a layer of denser texture; whilst in the shark, and other fish, the tooth is coated with a dense, shining, enamel-like substance, which is not, however, true enamel, nor the product of a distinct organ, but is developed from the same matrix as the rest of the tooth, is formed by the calcification of its external layer, and differs from the subjacent substance only in the greater proportion of the earthy particles, their more minute diffusion through the gelatinous basis, and the more parallel arrangement of the calcigerous tubes. In the sargus and balistes (file-fish) the dentine is harder, and is covered with a thick layer of a denser substance, developed by a distinct organ, and standing in the same relation with the enamel of higher animals, although differing from it (as already noticed,) in its minute structure, which approaches more nearly to that of dentine. The ossification of the capsule of the matrix gives to the enamel of the teeth of the file-fish, and some others, a thin coating of a third substance, analogous to the "cementum" of mammalian teeth. And in the pharyngeal teeth of the parrot-fish, a fourth substance is added to the structure of the tooth, by coarser ossification of the pulp, after its peripheral portion has been converted into the dense ivory. The teeth, thus consisting of dentine, enamel, cement, and coarse bone, are the most complicated as regards their substance, that have been yet discovered.

Four principal modifications are described by Prof. Owen as existing in the tubular structure of the dentine of fishes; and of these we shall extract his account.

"I. Premising that the essential character of this structure is a *cavitas pulpi*, or medullary canal, from which the calcigerous tubes radiate, the first modification which may be noticed is where the tooth is traversed by a number of equidistant and parallel medullary canals, each canal and its system of medullary tubes representing a cylindrical or prismatic denticle, and being separated from the contiguous denticles by a thin coat of bone or cement. This modification is exemplified in the rostral teeth of the saw-fish (*Pristis*,) the tesselated teeth of the eagle-rays (Myliobates, &c,) and the maxillary plates of the chimæroids. The dense dental case of the jaws of the parrot-fishes may likewise be regarded as an extreme instance of this modification; and we shall find the same structure reappearing in one of the inferior genera of the mammalian class. In the parrot-fishes, the denticles are quite distinct from one another; but in the saw-fish, chimæra, and eagle-rays, the contiguous medullary canals occasionally anastomose together. In the chimæroid fishes, these anastomoses are more numerous, and the boundaries of the component denticles less distinct; so that they form a transition between the preceding and what may be regarded as the second variety of the tubular structure.

"II. In this modification, the substance of the tooth is traversed by medullary canals, somewhat less regularly equidistant and less parallel than in the first; having the boundaries of their respective systems of radiated calcigerous tubes indicated by the minute calcigerous cells, with which the terminal branches of those tubes communicate; these boundaries being more or less obscured by the terminal branches of the calcigerous tubes extending across into the interspaces of the corresponding branches of an adjoining system of tubes, and anastomosing with them immediately, or through intervening dilatations or cells. The medullary canals here dichotomize more frequently than in the first modification; their anastomoses are more numerous, and the whole tooth, which is generally of large size, is consequently more individualized and compacted. The teeth of the Port Jackson shark (*Cestracion Philippi*) afford a good example of this modification, which also prevails in those of the extinct genera *Ptychodus*, *Psammodus*, &c. In the teeth of the extinct Acrodus, the medullary canals, which likewise traverse in great numbers the body of the tooth, assume a more or less wavy course; and this disposition, combined with their numerous anastomoses, leads to the third modification, which at the same time is the most common and characteristic of the dental structure in the class of fishes.

"III. In teeth manifesting this variety of the tubular structure, the dentine is perforated by a network of medullary canals, of which the interspaces are occupied by the calcigerous tubes and cells. The medullary canals are directly continued from those of the common bone with which the base of the tooth is anchylosed, or into which it has been converted. As the medullary canals proceed through the tooth, they maintain a course more or less parallel, and more or less straight, or wavy; but they ramify abundantly, and gradually diminish in calibre as they approach the surface of the tooth.....

"IV. The last type of structure is that which characterizes the teeth of most reptiles and mammalia. Here the dentine consists of a single medullary or pulp-canal, and a single system of calcigerous tubes radiating from the central or sub-central canal, at right angles to the periphery of the tooth. The crowns of the teeth of the extinct sauroid fishes and pycnodonts, the maxillary teeth of the existing file-fishes (*Balistes*) and angler (*Lophius,*) the incisors, canines, and molars, of the breams or sparoid fishes, the pharyngeal pavement of the wrasse-tribe (*Labridæ,*) the maxillary and pharyngeal denticles of the parrot-fishes (*Scari,*) and the lamelliform denticles of the crop-fishes (Diodon and Tetrodon,) likewise the maxillary teeth of some of the genera of sharks and rays, afford examples of this structure." (p. 13.)

Now of these four kinds of structure, the second and third are peculiar to the class of fishes; so that a tooth or even the minute fragment of a tooth, which shall be found to exhibit either of these, may be unhesitatingly referred to the piscine class. The first modification, also, is peculiar to fishes; with the exception of a single mammalian genus, Orycteropus. The fourth variety is regarded as belonging to a higher type, because it is characteristic of the teeth of vertebrate animals, which are higher in the scale than fishes; but if the grade of organization of a tooth be rated according to the proportion of vascular substance and vital power which it possesses, then those teeth which most resemble bone should be regarded as the most highly organized; and such are the teeth most common in fishes. In no teeth, however, is the dentine so dense, as in those of certain fishes, especially the scarus and diodon, which have been cited as examples of the fourth modification of the dentinal structure. These teeth strike fire with steel; yet they present an organized structure of minute complexity; and the tubuli are nowhere so numerous, so minute, so beautifully ramified and interlaced together.

The *development* of the teeth of fishes presents several points of great physiological interest. Its history is always the same, up to a certain point, with that of the development of the teeth in higher animals; but the process, in many instances, does not extend beyond the earlier and simpler stages observable in the latter. We cannot forbear extracting nearly the whole passage in which Prof. Owen sets forth the generalities of this subject:

"In all fishes, as in other vertebrate animals, the first step is the production of a simple papilla from the free surface of either the soft external integument, as in the young *pristis* (sword-fish,) or of the mucous membrane of the mouth, as in the rest of the class. In these primitive papillæ, there can be very early distinguished a cavity containing fluid, and a dense membrane (*membrana propria pulpi*) surrounding the cavity, and itself covered by the thin external buccal mucous membrane, which gradually becomes more and more attenuated as the papilla increases in size. In some fishes, as the sharks and rays, the dental papillæ do not sink into the substance of the vascular membrane from which they grow, but become buried in depressions of an opposite fold of the same membrane; these depressions enlarging with the growth of the papillæ, and forming the cavities or capsules in which the development of the tooth is completed. They differ from the capsules of the matrix of the mammiferous tooth, in having no organic connexion with the pulp, and no attachment to its base; the teeth, when fully formed are gradually withdrawn from the above described extraneous capsules, to take their place and assume an erect position on the alveolar border of the jaws." (p. 15.)

Here, therefore, is represented on a large, and, as it were, persistent scale, the *first* and transitory *papillary* stage of the development of the mammalian teeth; and the simple crescentic cartilaginous maxillary plate, with the mucous groove behind it containing the germinal papillæ of the teeth, offers in the shark a magnified representation of the earliest condition of the jaws and teeth in the human embryo.

"In many fishes, as the lophius and pike, the dental papillæ become buried in the membrane from which they arise; and the surface to which their basis is attached becomes the bottom of a closed sac. But this sac is never lodged in the substance of the jaw, the development of the tooth being completed in the tissue of the thick and soft gum or mucous membrane from which the papillæ were originally developed; hence teeth in various stages of growth are frequently brought away with that membrane when it is reflected from the jaw-bone. The ultimate fixation of the teeth, so formed, is effected by the development of ligamentous fibres in the submucous tissue between the jaw and the base of the tooth; which fibres become the medium of connexion between those parts, either as elastic ligaments, or by continuous ossification." (p. 16.)

Here, then, we meet with the *second* or *follicular* stage in the development of the mammalian tooth; the pulp being imbedded in a follicle of the mucous membrane; but the eruptive stage of the tooth takes place without any previous inclosure of the follicle and pulp in the substance of the jaw.

"In the *balistes*, *sparoids*, *sphyræna*, *scarus*, and many other fishes, the formation of the teeth presents all the usual stages which have been observed to succeed each other in the dentition of the most highly-organized animals; the papilla then sinks into a follicle, becomes surrounded with a capsule, and is then included in a closed alveolus of the growing jaw, where the development of the tooth takes place, and is followed by the usual eruptive stages." (p. 16.)

Thus, even in fishes, we have examples of the most complete form of dental development; as might, indeed, have been anticipated from the fact that the teeth, in some of this class, present the union of all the structures which are met with in the superior tribes. It is obvious that where the development ceases at the papillary stage, as in the shark, there *can* be no distinct enamel-pulp; but in every case in which the development goes on to the follicular stage, an enamel-pulp *may* be developed. It is seldom very conspicuous, however; and the substance into which it is converted differs considerably, as formerly stated, from the enamel of the teeth of higher animals, and bears a much greater analogy to the dentine. The cementum, where it is present, is developed on precisely the same plan as in higher animals; namely, by the ossification of the outer layer of the capsule.

In all fishes, the teeth are being continually shed and renewed; the only teeth which can be called permanent being those in the rostrum of the saw-fish; and even these being capable of being properly regarded, in Prof. Owen's opinion, as modified dermal spines. Where the first teeth are developed in alveolar cavities, the succeeding ones follow them in the vertical direction; and owe the origin of their matrix to the continuation, from the mucous capsule of their predecessors, of a cæcal process, in which the papillary rudiment of the dental pulp is developed, just as, according to Mr. Goodsir's researches, the follicles of the human permanent teeth are formed as diverticula from those of the deciduous. But in the great majority of fishes, the germs of the new teeth are being continually developed, as those of the old originally were, from the free mucous membrane of the mouth,—a condition which is peculiar to the present class. The angler, the pike, and many of our common fishes illustrate this mode of dental reproduction. But it is peculiarly conspicuous in the cartilaginous fishes, in which the entire phalanx of their numerous teeth is ever moving slowly forward, in a sort of rotatory progress over the alveolar border of both upper and under jaws; the teeth being successively cast off as they reach the outer margin, and new teeth rising up in equal proportion from the mucous membrane behind the rear rank of the phalanx. This endless succession of new and sometimes highly-complicated matrices, could hardly seem otherwise than a waste, as Prof. Owen justly observes, to those who hold the doctrine that the process of dental development is one of "transudation" or "excretion," and who regard the pulp in the light of a gland; whilst it is the necessary consequence of the "conversion" of the pulps into teeth, that as fast as the latter are to be shed and replaced, new pulps should be produced to supply them.

For more minute details regarding the differences of form and structure, which characterize the teeth of the several subdivisions of the class, we must refer to the 'Odontography' itself; contenting ourselves with adducing a very interesting example of their value, as serving to determine the real nature of fragments which present themselves in a fossil state. Two fossils, consisting of portions of jaws with teeth of a simple conical form, and evidently belonging to the same or to nearly allied genera, though of distinct species, had been described by two eminent American naturalists, Dr. Harlan and Dr. Isaac Hays, as extinct members of the Saurian order of reptiles; Prof. Agassiz, however, was led, from the external characters of the jaws and teeth in question, to believe that they might belong to the Scomberoid (mackerel) family in the class of fishes. Having examined the microscopic structure of the teeth, Prof. Owen found that they were traversed by medullary tubes; of which the larger maintained a nearly parallel longitudinal course throughout the body of the tooth, and were united by smaller transverse branches; the spaces between these everywhere exhibiting plexiform groups of flexuous calcigerous tubes, proceeding from the medullary canals. Now it has been already stated, that this peculiar distribution of the medullary canals in teeth is restricted to fishes; the idea of the reptile character of these remains was therefore altogether untenable. Further, the particular modifications of this structure presented by the teeth in question, are most closely allied to those which characterize the teeth of the Scomberoid fishes; so that justice of the determination made by Prof. Agassiz was by this mode of investigation fully confirmed.

We now pass on to the class of Reptiles; in certain groups of which, as is well known, teeth are altogether wanting. In the tortoises and turtles, the jaws are covered by a sheath of horn, which in some species is of considerable thickness and very dense; its working surface is trenchant in the carnivorous species, but variously sculptured, and adapted both for cutting and bruising, in the vegetable-feeders. That this substance really has a greater analogy with the teeth which it replaces, than would be at first sight imagined, appears from the history of its development; for this commences (as in the parrot-tribe among birds) from a series of distinct papillæ, which sink into alveolar cavities, regularly arranged (in *trionyx*) along the margins of the upper and lower jaw-bones; these alveoli are indicated by the persistence of vascular canals long after the originallyseparate tooth-like cones have become confluent, and the horny sheath completed. The toads, among the Batrachia, afford another example of the absence of teeth; and here there is no compensating structure. In the Saurian order, there is no exception to the general rule of the presence of teeth; and among the serpents there is but one, the *Coluber scaber.* In this animal, there is a very remarkable adaptation of other parts, to make up for the deficiency; the inferior spinous processes of certain of the cervical vertebræ are unusually prolonged, and penetrate the coats of the œsophagus; and their extremities, which are thus introduced into the alimentary canal, are coated with a layer of hard dentine, and form substitutes for the true teeth, which, if not always entirely absent, are merely rudimental in the ordinary situations in the mouth.

The *number* of teeth in reptiles is usually greater than in mammalia, and less than in fishes; the smallest known number, where teeth exist at all (except in the case of the anomalous Dicynodon, p. 403), is in certain Monitors, which have thirty teeth; and certain Batrachians, which have upwards of eighty teeth in each lateral maxillary series, present the largest number. It is interesting to observe that the number is the greatest and least definite in those reptiles, which present the nearest approach to fishes; and that it diminishes, and shows a nearer approach to definiteness, in the higher parts of the series. The number of teeth, however, is rarely so fixed and determinate in any reptile, as to be characteristic of the genus or species.

In regard to *situation,* also, the teeth of reptiles are intermediate between those of fishes and mammals. They are frequently present in the jaws only, as in the crocodiles and many lizards; but in several instances, especially among the batrachia, they are present also upon the roof of the mouth, being implanted in the palatine and pteregoid bones, the vomer and sphenoid; and these last are usually arranged in several rows, like the 'dents en cardes' of fishes. The teeth of reptiles are for the most very simple as to *form;* being generally of a nearly conical shape, with the crown more or less curved, and the apex more or less acute. Thus they are adapted rather for seizing and holding the prey, than for dividing and masticating it. The greatest departure from the usual form is seen in the herbivorous Iguana, and the gigantic extinct Iguanodon which resembled it in habit; in the teeth of these animals, the crown is expanded, and the margins are notched; and those of the Iguanodon are further marked by longitudinal ridges, presenting the most complicated external form ever met with in this class. In no reptile does the base of the tooth ever branch into fangs; which is a character of great importance in the distinction of the teeth of reptiles from those of mammals.

The mode of *attachment* of the teeth of reptiles presents several varieties, which are extremely important in zoological classification, and in the recognition of fossils. When the teeth remain distinct from the jaw, they may be lodged either in a continuous groove, as in the Icthyosaurus, or in separate sockets, as in the Plesiosaurus and Crocodilians. As a general rule, however, they are anchylosed, at some point, to the bone which supports them; and it is in the place and manner of the anchylosis, that the differences exist which have been just alluded to.

"The base of the tooth is anchylosed to the walls of a moderately deep socket in the extinct megalosaur and thecodon. In the labyrinthodons and cæciliæ among the batrachians; in most ophidians; and in the geckos, agamians, and varanians, the base of the tooth is imbedded in a shallow socket and confluent therewith. In the scincoidians, safeguards, in most iguanians, in the chameleons and most other lacertian reptiles, the tooth is anchylosed by an oblique surface extending from the base more or less upon the outer side of the crown, to an external alveolar plate of bone; the inner alveolar plate not being developed. In the frogs, the teeth are similarly but less firmly attached to an external parapet of bone. The lizards which have the teeth thus attached to the side of the jaw, are termed 'pleurodonts.' In a few iguanians, the teeth appeared to be soldered to the margins of the jaws; these have been termed 'acrodonts.' In some extinct lacertians, as the mososaur and leiodon, the tooth is fixed upon a raised conical process of bone.

"These modifications of the mode of attachment of the teeth of reptiles are closely adapted to the destined application of those instruments and to the habits of the species; we may likewise perceive that they offer a close analogy to some of the transitory conditions of the human teeth. There is a period, for example (at the sixth week), when the primitive dental papillæ are not defended by either an outer or an inner alveolar process, any more than their gigantic calcified analogues in the extinct mososaur. There is another stage (at the seventh or eighth week,) in which the groove containing the dental germs is defended by a single external cartilaginous alveolar ridge; this condition is permanently typified in most existing lizards. Next there is developed an internal alveolar plate, and the sacs and pulps of the teeth sink into a deep but continuous groove, in which traces of transverse partitions soon make their appearance; in the ancient icthyosaur, the relation of the jaws to the teeth never advanced beyond this stage. Finally, the dental groove is divided by complete partitions (at the sixth month,) and a separate socket is formed for each tooth; and this stage of development is attained in the highest organized reptiles, as the crocodile." (p. 183.)

The *substance* of the teeth of reptiles usually consists for the most part of dentine and cement; these alone are present in the teeth of the Batrachian and Ophidian reptiles, in which a thin layer of cement invests the central body of dentine, and follows any inflections or sinuosities that may characterize the surface of the latter. In the Saurians, the crown of the tooth is usually defended by a thin coat of enamel; whilst the interior is not unfrequently filled up by bone, formed by the ossification of the last remains of the pulp,—which may take place not merely in the teeth which are anchylosed to the jaw, but also in some teeth, as those of the Icthyosaurus, which remain free.

The varieties of dental *structure* are few in the reptiles, as compared either with fishes or mammals; and its most complicated condition arises from the interblending of the dentinal and other substances, rather than from modifications of the tissues themselves. In the teeth of most reptiles, the intimate structure of the dentine corresponds with that which has been described as its fourth type or modification in the teeth of fishes, and which is the prevailing structure of mammalian dentine, viz. the radiation of a system of minute calcigerous tubes from a single pulp-cavity, at right angles to the external surface of the tooth. The most essential modification of this structure is the intermingling of cylindrical processes of the pulp-cavity, in the form of medullary canals, with the finer tubular structure; as is seen in the Iguanodon. These radiations from the central medullary cavity cannot, however, be confounded with the reticulated arrangement of the medullary canals in the teeth of fishes; for they run, at pretty definite intervals, through the dentine, parallel with the calcigerous tubes, which do not arise from them, but (as in the teeth of other reptiles) from the central medullary cavity alone. A similar arrangement is to be found in the coarse dentine, which characterizes the teeth of the Sloth and Megatherium, animals formed, like the gigantic Iguanodon, for a vegetable diet. The cementum is simply and minutely cellular upon the crowns of the teeth of reptiles; but it exhibits the radiated cells at the base of the tooth in the anourous Batrachians and in Saurians. The enamel is sub-transparent, dense, and minutely fibrous, in all the reptiles which have their teeth defended by this substance.

Some very curious modifications present themselves in the teeth of certain reptiles, as a consequence of a sort of folding of the dentine and its envelope of cement. A simple example of this is seen in the poison-fang of the venomous serpents; which, as is well known, is furnished with a canal that passes through the centre of the tooth at its lower part, and forms a groove along its side nearer the extremity. To form this canal, the two margins of the tooth are folded, as it were, towards each other; where they meet, they will completely inclose the canal, so as to convert it into a tube; and where they do not meet, they will constitute a groove. Notwithstanding this singularly-modified disposition of the dentine, which is thus converted at the lower part of the tooth into a hollow cylinder, it still retains its normal structure; and although the pulp-cavity is reduced to the form of a crescentic fissure, the calcigerous tubes continue to radiate from it, according to the usual law. A similar change is carried to a far greater extent, in the tooth of an extinct reptile, which has received from Prof. Owen the appropriate title of Labyrinthodon, from the extraordinary peculiarities of its dental structure. Of these peculiarities we despair of giving our readers an adequate idea without the aid of a figure; since they produce the most complicated structure that has yet been encountered in the tooth of any animal. We must request them in the first place to conceive of the conical tooth, with a central pulp-cavity, as having its substance composed of a number of vertical plates, arranged in a radiating manner. Each of these plates is composed of dentine, arranged on the two sides of a prolongation or 'medullary ray' of the central pulp-cavity; and from this prolongation the tubuli of the plate of dentine originate on either side. On the other hand, the capsule sends prolongations from the exterior of the tooth, which pass between the contiguous plates; and the ossification of these gives to each plate a coating of cement, along its whole surface. Now let us suppose that all these plates, instead of having plane surfaces, were thrown into cerebriform convolutions; still preserving, however, their general radiating direction; we shall then have an idea of the arrangement of the parts of the tooth of the Labyrinthodon. The links by which this complex arrangement are connected with simpler modes of conformation, are seen in the base of the teeth of the Icthyosaurus, and in the Lepidosteus, a sauroid fish of the present epoch. The remarkable elegance of this structure, when displayed by a transverse section of the tooth, is evidenced by the fact, that within a short time after the publication of the Second Part of the Odontography, a pocket-handkerchief was purchased in Manchester, bearing an enlarged copy of the section of the Labyrinthodon tooth as its pattern. We are sure that if our manufacturers and designers would more frequently have recourse to nature for suggestions, the progress of their arts would be greatly accelerated.

We shall have a little more to say of the Labyrinthodon presently; but must first extract Prof. Owen's general remarks in regard to the *development* of the teeth of reptiles.

"The teeth of reptiles are never completed, as in certain fishes, at the first or papillary stage; but the pulp sinks into a follicle, and becomes inclosed by a capsule: the process of development, however, never offers the eruptive stage, in the sense in which this is usually understood, as signifying the extrication of the young tooth from a closed alveolus.

"The completion of a tooth is soon followed by preparation for its removal and succession; the faculty of developing new tooth-germs seems to be unlimited in the present class, and the phenomena of dental decadence and replacement are manifested at every period of life: the number of teeth is generally the same in each successive series, and the difference of size presented by the teeth of different and distant series is considerable.

"The new germ is always developed, in the first instance, at the side of the base of the old tooth, never in the cavity of the base; the crocodiles form no exception to this rule. The poison-fangs of serpents succeed each other from behind forwards; in almost every other instance, the germ of the successional tooth is developed at the inner side of the base of its predecessor.

"In the frog, the dental germ makes its appearance in the form of a papilla, developed from the bottom and towards the outer side of a small fissure in the mucous membrane or germ, that fills up the shallow groove at the inner side of the alveolar parapet and its adherent teeth; the papilla is soon enveloped by a capsular process of the surrounding membrane: there is a small enamel-pulp developed from the capsule opposite the apex of the tooth; the deposition of the earthy salts in this mould is accompanied by ossification of the capsule, which afterwards proceeds *pari passu* with the calcification of the dentinal papilla or pulp; so that, with the exception of its base, the surface of the uncalcified pulp alone remains normally unadherent to the capsule. As the tooth acquires hardness and size, it presses against the base of the contiguous attached tooth, causes a progressive absorption of that part, and finally undermines, displaces, and replaces its predecessor. The number of nascent matrices of the successional teeth is so great in the frog, and they are crowded so closely together, that it is not unusual to find the capsules of contiguous tooth-germs becoming adherent together as their ossification proceeds. After a brief maceration, the soft gum may be stripped from the shallow alveolar depression; and the younger tooth-germs in different stages of growth are brought away with it." (p. 186.)

The mode of development of the teeth of serpents does not differ essentially from that of the teeth of the bratrachian above described; except in the relation of the papilla of the successional poison-fangs to the branch of the poison-duct that traverses the cavity of the loose mucous gum in which they are developed: this relation has not yet been clearly developed. We should be disposed to think that a new branch of the duct is developed for every papilla; rather than that the new poison-fang can be brought into the same relation with the severed duct of the poison-gland, as the displaced fang which it succeeds.

In the greater number of the lizard tribe, the phenomena of dental development nearly coincide with those which have been just detailed in regard to the frog; the successional teeth being developed on the inner side of the old ones, and coming to press upon and replace the others.

"In the Acrodont lizards, and those in which the teeth are anchylosed to the summits of bony processes, the successional teeth are in like manner developed at the inner side of the supporting processes, gradually penetrate them as their growth proceeds, and finally undermine and displace the tooth, and become in their turn anchylosed to new bony eminences of the alveolar tract. The jaws of the gigantic Mososaur exhibit on a large scale different stages of this mode of shedding and replacement, which is so general in the class of reptiles.

"In the Icthyosaurus, in which, by the development of an internal as well as an external alveolar plate, the teeth are lodged in a deep continuous groove, the successional germs were also developed in this extinct reptile at the inner side of their predecessors; and from the solidification of the implanted base of the fully-formed tooth, occasioned an extensive absorption of its inner side, before it finally yielded to the lateral pressure.

"In the Crocodile, the tooth-germ is developed from the vascular membrane covering the base of the internal wall of the socket; it is soon invested by a capsule, and by its pressure causes the formation of a shallow recess, or secondary alveolus, in the contiguous bone. In this alveolus, however, it never becomes inclosed like the successional teeth in most Mammalia; for, exerting equal pressure against the fang of the contiguous tooth (which, from being incompletely formed, has a wide pulp-cavity with very thin walls), the nascent tooth soon penetrates that cavity, and quits the recess in the alveolar plate in which it was originally situated. Thus the stage of development corresponding with the 'eruption' of the tooth in the mammalia, is immediately followed by the 'inclusion' of the new tooth in the pulp-cavity of its predecessor. . . . . The rapid succession of tooth-germs, which stamps the impress of decay upon their predecessors often before the growth of these is completed, though common to many reptiles, is most strikingly manifested in the crocodiles; in which three and sometimes four generations of teeth, sheathed one within the other, are contained in the same socket." (p. 187.)

The Saurian group furnishes one singular exception to the general rule which we have seen to prevail through the reptile class, in regard to the want of permanence in the individual teeth; this is furnished by the *Dicynodon*, an extinct Saurian of South Africa, which had in the upper jaw two long canine tusks, that must have grown and been maintained through life of due size and strength, like the tusks of the boar and walrus. With the exception of these two anomalous teeth, the jaws of the Dicynodon were edentulous.

We cannot adduce a better example of the extensive applicability of the microscopic test, than the exact determination which it enabled Prof. Owen to make, of the identity in character between certain teeth found in two different and remote deposits,—the consequent settlement of the question as to the relation of equivalence between these deposits,—and the extraordinary results to which he was conducted by the investigation thus commenced, as to the former existence of a gigantic animal of the Frog kind, by whose feet those imprints were made in the sand, which, preserved in the hardened sands without any other traces of the animal that made them, have been a source of so much perplexity to geologists. The first-discovered fossils belonging to the Batrachian genus now termed Labyrinthodon (from the extraordinary structure of the teeth, already described) were certain detached teeth, found in the keuper sandstone of Wirtemberg, and described by Prof. Jaeger as the remains of some gigantic Saurian reptile, to which he gave the name of Mastodonsaurus. Other fossil fragments of jaws and teeth, from the same formation, and believed by Prof. Owen to be referrible to the same genus, were described under the name of Phytosaurus. A third remarkable and characteristic fossil, discovered in the keuper sandstone, consists of the occipital portion of the cranium with two large and separate condyles, as in the Batrachian reptiles; and on this fossil Prof. Jaeger founded his species called "Salamandroides giganteus." Now in a sandstone deposit in Warwickshire, certain teeth or fragments of teeth, at first supposed to be of Saurian character, were discovered a few years since; and a question having been raised whether this sandstone was the equivalent of the keuper or the bunter division of the new red-sandstone formation, as developed in Germany, it became a matter of great scientific importance to determine whether the Warwick and Wirtemberg fossil teeth were specifically or generically identical, or whether they were altogether dissimilar. From what has been already stated as to the general correspondence of the teeth of reptiles with one type of form, it will not be surprising that Prof. Owen found himself unable to pronounce upon this question, from external characters; it only remained to resort to the test of intimate structure; and on this he did not found any great hopes.

"Hitherto in investigating the intimate texture of the teeth of Saurian reptiles, I had found the dentine or body of the tooth to consist of the finest calcigerous tubes, radiating according to the usual law, from the pulp-cavity, at right angles to the external surface of the tooth, which is covered by a simple investment of enamel; from the prevalence of this structure in the simple conical teeth of reptiles, I did not build any very strong hopes of detecting such modifications of dental structure in the similarly simple teeth of the so-called Mastodonsaurus, and of the tooth from the Warwick sandstone, as would be sufficiently marked and obvious to carry conviction of their generic, much less specific identity. But in this I was agreeably and unexpectedly deceived.

"When I refer to fig. 1, in Plate 64 A, and state that the first transparent transverse section of the tooth of the Labyrinthodon (Mastodonsaurus) Jaegeri that was placed under the microscope and viewed by transmitted light with a low magnifying power, presented the singularly complicated structure there exhibited, the anatomist, conversant with the known modifications of dental structure in the animal kingdom, may well conceive my surprise. It was not, indeed, until I had had sections made in various directions, from the portions of the tooth of the Labyrinthodon Jaegeri transmitted to me, and had studied them intently at several successive examinations, comparing the appearances they presented with those of numerous examples of the teeth of Saurians, Batrachians, and other animals, that I at length comprehended the nature and principle of the singular cerebriform convolutions or sinuosities, which pervaded every portion of the tooth of this most remarkable reptile of the keuper sandstone." (p. 200.)

The next step was, of course, to make a similar examination of the teeth from the Warwickshire sandstone; the results of which were such as to leave no doubt of their very close alliance to those just alluded to; all the peculiarities of this most extraordinary type of dental structure being so closely preserved in the British specimen, as to leave no doubt of its generic identity at least with the German,—the differences being such as might be reasonably expected in distinct species of the same genus. Thus, therefore, the question was settled, so far as its immediate geological bearing was concerned; since the existence of similar remains, of so very singular a character, in the two deposits in question, was sufficient with other evidences to prove their equivalence. But a most interesting zoological question then arose, as to the nature of the animal to which these remains belonged. As already mentioned, they had been referred, from *external* characters, to the Saurian order; but these characters were by no means conclusive, owing to the general similarity in form, which prevails through the teeth of the entire class. The nearest approach, among reptiles, in the *internal* structure of the teeth, is presented by the Icthyosaurus; in which the characters of the Saurian head and teeth are combined with Icthyic vertebræ and extremities. A nearer approach was presented in the teeth of the Lepidosteus which possesses, with the general conformation of a fish, a bifid air-bladder resembling the lungs of reptiles, and other characters of elevation towards the Sauria. Hence it might be expected, that the labyrinthodon would combine with its reptile characters an affinity with fish; and this idea was fully borne out by the examination of fragments of the skull of the Warwick fossil, with which the teeth remain in connexion; from which it appeared to be an unquestionable result, that the labyrinthodon, with many affinities to the crocodilians, was in reality a gigantic batrachian with peculiar affinities to fishes. With this clue, there could be little hesitation in regarding the occipital fragment of the keuper sandstone, whose *batrachian* character had been recognized when first discovered, as a part of the animal to which the teeth belonged, —as representing, in fact, the very Labyrinthodon Jaegeri, whose teeth had been described as Saurian.

Of this curious genus, five species have been determined by Prof. Owen; the smallest of these far exceeding in dimensions the largest living species of the same order; and the larger ones measuring several feet in length. One of these appears to have had its skin covered, in part at least, with bony scutes resembling those of the crocodile; but as the skin, in all animals, is one of the most variable of organs, this character must not be regarded as indicative of any close affinity, since all the essential points of structure hitherto discovered agree in assigning to the animal a place among the tail-less batrachia. This is indicated by the very large proportionate size of the posterior extremities; which must have been three or four times the length of those of a crocodile of corresponding size as far as regarded the anterior portion of the body; but which would harmonise very well with the relative length of those of some of the living tail-less batrachians.

We have already alluded to certain foot-prints found in various localities in the new red sandstone, as having been a fertile source of geological speculation. These impressions indicate an animal remarkable for the disproportionate magnitude of its posterior extremities, and also for the singular shape of its foot; and it had been suggested by more than one palæontologist, that the impressions of the *cheirotherium* (which was the provisional name given to the unknown animal in question) were those of a batrachian. In consequence of certain peculiarities in these impressions, however, it was obvious that the animal must have been quite distinct in the form of its feet from any known batrachian or other reptile. Now we have seen that in the beds of the very same formation, we find also the teeth and bones of a batrachian reptile, of dimensions and proportions such as might produce the footsteps, and differing from all other batrachia —indeed from all other reptiles—in the structure of its teeth, and therefore (it may be fairly inferred,) in that of its extremities. When it is also borne in mind that both the fossils and the footprints are peculiar to the new red-sandstone, we think it will be admitted that a very strong case has been made out for the establishment of the identity of the Labyrinthodon and the Cheirotherium—or, in other words, for the belief that the foot-prints so remarkably preserved were made by the feet of the Labyrinthodon.

The evidence required for the conclusive establishment of this idea, may very probably be furnished ere long, by the discovery of the bones of the extremities of the labyrinthodon; the nature of which cannot be exactly predicated from the fragments at present known.

We must reserve the consideration of the teeth of Mammals, as well as what we intend to say of the researches of Dr. Carpenter and Mr. Bowerbank, to another occasion.

